# Cannabinoid Receptor 1 Gene Polymorphisms and Nonalcoholic Fatty Liver Disease in Women with Polycystic Ovary Syndrome and in Healthy Controls

**DOI:** 10.1155/2014/232975

**Published:** 2014-07-17

**Authors:** Justyna Kuliczkowska Plaksej, Lukasz Laczmanski, Andrzej Milewicz, A. Lenarcik-Kabza, Anna Trzmiel-Bira, Urszula Zaleska-Dorobisz, Felicja Lwow, Lidia Hirnle

**Affiliations:** ^1^Department of Endocrinology, Diabetology and Isotope Therapy, Wroclaw Medical University, 4 Pasteura Street, 50-367 Wroclaw, Poland; ^2^Department of Radiology, Wroclaw Medical University, 68 Curie-Sklodowskiej Street, 50-369 Wroclaw, Poland; ^3^Department of Health Promotion, University School of Physical Education, 35 Paderewskiego Street, 51-612 Wroclaw, Poland; ^4^First Department of Gynaecology and Obstetrics, Wroclaw Medical University, 3 Chalubinskiego Street, 50-368 Wroclaw, Poland

## Abstract

*Context*. Polycystic ovary syndrome (PCOS) is frequently associated with nonalcoholic fatty liver disease (NAFLD). The endocannabinoid system may play a crucial role in the pathogenesis of NAFLD. Polymorphism of the cannabinoid receptor 1 gene (*CNR1*) may be responsible for individual susceptibility to obesity and related conditions. *Objective*. To determine the role of genetic variants of * CNR1* in the etiopathology of NAFLD in women with PCOS. * Design and Setting*. Our department (a tertiary referral center) conducted a cross-sectional, case-controlled study. * Subjects*. 173 women with PCOS (aged 20–35) and 125 healthy, age- and weight-matched controls were studied. * Methods*. Hepatic steatosis was assessed by ultrasound evaluation. Single nucleotide polymorphisms of * CNR1* (rs806368, rs12720071, rs1049353, rs806381, rs10485170, rs6454674) were genotyped. * Results*. Frequency of the G allele of rs806381 (*P* < 0.025) and the GG genotype of rs10485170 (*P* < 0.03) was significantly higher in women with PCOS and NAFLD than in PCOS women without NAFLD. Frequency of the TT genotype of rs6454674 was higher in PCOS women with NAFLD (not significantly, *P* = 0.059). In multivariate stepwise regression, allele G of rs806381 was associated with PCOS + NAFLD phenotype. * Conclusion*. Our preliminary results suggest the potential role of * CNR1* polymorphisms in the etiology of NAFLD, especially in PCOS women.

## 1. Introduction

Polycystic ovary syndrome (PCOS) is one of the most common hormonal disorders in women in reproductive age, with a prevalence of 5–10% [1]. According to the Rotterdam criteria, its prevalence may be even as high as 18% [2]. PCOS is associated not only with alteration in sex hormones but also with metabolic disturbances such as abdominal obesity, insulin resistance (IR), atherogenic dyslipidemia, and diabetes mellitus (DM), all of them being characteristic features of metabolic syndrome (MS) [3]. Another manifestation of obesity is nonalcoholic fatty liver disease (NAFLD), with a prevalence of 70% in overweight and obese patients [4]. NAFLD prevalence is also increased in DM and dyslipidemia and, in turn, clinical features of MS are often observed in NAFLD patients [4–7]. Nowadays NAFLD is considered to be not only the hepatic manifestation of MS [8] but also an early predictor of metabolic disorders in the general population and a major cause of chronic liver disease in overweight and obese subjects [7]. Many data indicate that NAFLD and PCOS not only share features of MS but also are interconnected. There is a high proportion of NAFLD among PCOS women and, conversely, women with NAFLD often present many features of PCOS [5, 9, 10]. The exact etiopathology of both syndromes is still a matter of debate.

A possible mechanism that may underlie metabolic disturbances and features of PCOS and NAFLD may be dysfunction of the endocannabinoid system (EC). The EC plays a crucial role in energy homeostasis by modulation of appetite, food intake, and energy storage. Its action is transmitted by activation of two main types of receptors, 1 and 2 (CB1 and CB2), located not only within the brain but also in many peripheral tissues including the gastrointestinal tract, liver, skeletal muscles, pancreas, and adipocytes [11]. Dysregulation of EC has been observed in overweight, obesity, and eating disorders and may be involved in the pathogenesis of IR, NAFLD, and MS [12–16]. In obese patients higher levels of cannabinoids in comparison to lean patients were strongly associated with visceral obesity, dyslipidemia, and IR. Blockade of CB1 inhibits food intake, promotes weight loss, inhibits adipocyte proliferation, decreases IR and waist circumference, and improves lipid profile [17–21]. CB1 has been identified in the liver and the liver has been shown to produce endocannabinoids [12, 22, 23]. During liver pathology the EC is activated and CB1 and CB2 are markedly upregulated, most particularly in stellate cells and vascular endothelial cells of the cirrhotic liver [24]. In animal studies, a high-fat diet increases the hepatic level of cannabinoids, density of CB1, and fatty acid synthesis, which can be reduced by CB1 blockade [12, 22, 25, 26]. Activation of CB1 enhances experimental steatosis and a CB1 antagonist prevented the development of liver steatosis in rats [12, 27]. The relationship between splanchnic EC level and liver steatosis has been recently analyzed. Results of the study of Westerbacka et al. showed that the human liver takes up cannabinoid 2-arachidonoylglycerol and produces triacylglycerols, which might reflect increased lipogenesis [28]. Exogenous phytocannabinoids also affect the severity of steatosis [29]. Moreover, data derived from clinical trials strongly suggest that selective CB1 antagonism improves IR and reduces liver fat [30].

Recently, endocannabinoid receptors and cannabinoids were also discovered within human ovaries [31, 32]. CB1 and CB2 have been identified in the medulla and cortex of the ovary, in the granulosa cells of primordial, primary, secondary, and tertiary follicles, in the theca cells of secondary and tertiary follicles, and in the corpus luteum and corpus albicans. The EC is probably involved in the maturation of follicles and oocytes [31, 33]. Cannabinoids are able to modulate the function of the hypothalamic-pituitary-gonadal axis and downregulate blood luteinizing hormone levels, by indirectly modifying gonadotropin-releasing hormone secretion in humans [34]. A direct adverse effect of cannabinoids on the ovary has been clearly documented. The role of the EC in modulation of energy balance and metabolism control could also suggest an interaction with gonadal function. Obesity and IR are associated with menstrual irregularities, chronic anovulation, and infertility. There is also a functional EC in the pancreatic islet cells and cannabinoids are released concurrently with glucose-induced insulin secretion. Some studies show a negative effect of the EC on insulin secretion while others indicate the opposite [35–38]. It is possible that the local effect of endocannabinoid signaling in the pancreas might also play a role in PCOS associated IR [32, 39]. Results of some studies have suggested that there might exist several specific, still undefined dysfunctions of the EC associated with higher prevalence of obesity and IR, which in turn are implicated in pathological conditions such as PCOS [40, 41].

We hypothesized that polymorphic variants of the CB1 gene (*CNR1*) might be associated with differences in EC activity and function and potentially contribute to individual susceptibility to obesity and related complications. The aim of this study was to investigate whether common polymorphic variants of* CNR1* (rs806368, rs12720071, rs1049353, rs806381, rs10485170, and rs6454674) are associated with NAFLD frequency in PCOS women in comparison to healthy, age- and weight-matched controls.

## 2. Material and Methods

### 2.1. Subjects

The study group consisted of 173 women with PCOS, aged 20–35, diagnosed according to Rotterdam diagnostic criteria (2003), on the basis of two of the following features: (1) oligo- or amenorrhea, (2) clinical or biochemical hyperandrogenism, and (3) polycystic ovary in pelvic ultrasonography. Patients were excluded in case of: (1) having other causes of menstrual irregularity, (2) being pregnant, and (3) having other causes of hyperandrogenism such as hypercortisolism and 21-hydroxylase deficiency. A group of 125 healthy, age- and weight-matched controls was randomly selected from the Wroclaw city population. Women from the control group had a history of regular menstrual cycles and no evidence of hyperandrogenism. None of the women from the entire study population were on a special diet, suffered from chronic, systemic illness, smoked cigarettes, abused alcohol, used medications that influence lipid and glucose metabolism or influence liver function, or used oral contraceptive pills. Women with previously diagnosed chronic liver disorders such as viral hepatitis B and C, autoimmune hepatitis, or cirrhosis were excluded from the study. Ultrasonography of the liver and liver enzyme activity evaluation (serum aspartate and alanine aminotransferases: AST, ALT) were performed in all patients. The study group (PCOS) and control group were subdivided depending on the presence of NAFLD. All patients were informed about the aim and methods of the study and gave their written informed consent. The study protocol was approved by the Ethics Committee of Wroclaw Medical University.

### 2.2. Genetic Studies

Whole genomic DNA was isolated from blood leukocytes using standard methods.* CNR1* genotyping (rs12720071, rs1049353, rs806368, rs806381, rs10485170, and rs6454674) was performed by two multiplex polymerase chain reactions (PCR) and minisequencing.

The first one: three fragments of the* CNR1* gene (347-bp, 346-bp, and 231-bp) were amplified using multiplex PCR mix containing the specific three pairs of primers (see Table 1), 1x PCR buffer, 1.5 mM MgCl_2_, 200 *μ*M dATP, 200 *μ*M dCTP, 200 *μ*M dGTP, 200 *μ*M dTTP, 1x Q solution, 2 polymerase units (TAKARA), 200 ng genomic DNA, and water for a total volume of 20 *μ*L.

The DNA was denatured at 95°C for 3 minutes followed by 35 cycles of denaturation at 95°C for 30 seconds, annealing at 58°C for 30 seconds, and extension at 72°C for 45 seconds.

To amplify the second group of the four fragments of the* CNR1* gene (205-bp, 230-bp, 280-bp and 304-bp) multiplex PCR mix was used. It was employed containing: the specific four pairs of primers (see Table 4), 1x PCR buffer, 1.5 mM MgCl_2_, 200 *μ*M dATP, 200 *μ*M dCTP, 200 *μ*M dGTP, 200 *μ*M dTTP, 2 hot-start polymerase units (TAKARA), 200 ng genomic DNA, and water for a total volume of 20 *μ*L. The DNA was denatured at 95°C for 3 minutes followed by 35 cycles of denaturation at 95°C for 30 seconds, annealing at 56°C for 30 seconds, and extension at 72°C for 45 seconds.

The amplified fragments were purified from oligonucleotides and free dNTPs by SAP and ExoI treatment (Fermentas).

The minisequencing method was based on the incorporation of single fluorescence-labeled dideoxynucleotides to the 3′ end of the oligonucleotide that was correctly paired to the specific template DNA fragment using a SNaPshot kit (Applied Biosystems). Two SNaPshot reactions were carried out using the oligonucleotides:

- rs12720071: 5′-CTTGTTATGGTAGAAAAATTTCACG-3′
- rs1049353: 5′-TGCAGCCAGTGTTCACAGGGCCGCAGAAAGCTGCATCAAGAGCAC-3′
- rs806368: 5′-TTAAGATGCCACGGCAATGTAAAGAAACTCTCCCA-3′
- rs806381: 5′-TCCAACAAATGAGTGACCGTTACC-3′
- rs10485170: 5′-ACTAGAGTTGTGCTGAGTTAATACATGAGATC-3′
- rs6454674: 5′-CTTCTCCAAAATATTTCCTGGAATAAAAGAAGCAATAACT-3′

designed so that it ended immediately before the polymorphic side. The SNaPshot reaction consisted of 25 cycles: denaturation at 96°C for 10 seconds, annealing at 50°C for 5 seconds, and extension at 60°C for 30 seconds. The product was analyzed by an ABI 3100 sequencer (Applied Biosystems). Product sizes were calculated using GeneScan 4.1 (Applied Biosystems).

### 2.3. Ultrasound Evaluation

Ultrasonography of the liver was performed in the morning, between 8 and 9 a.m., after an overnight fast of at least 8 h. The mean duration of each examination was 20 minutes, which included assessment of the gall bladder, liver, and pancreas specifically and the rest of the abdomen. Diagnosis and stage of NAFLD were assessed according to the study of Saverymuttu et al. and Ma et al.: grade I (mild): increased echogenicity of liver compared with renal cortex or spleen; grade II (moderate): obscure hepatic and portal vein walls; grade III (severe): impaired visibility of the diaphragm [42, 43]. NAFLD was also diagnosed on the basis of elevated liver enzyme activity defined as values above the upper limit of normal in our department laboratory (>35 U/L).

### 2.4. Statistical Analysis

The prevalence of genetic polymorphisms was analyzed in compliance with the law of Hardy-Weinberg. Genotype distribution was determined using the http://www.e-laboratorium.com.pl website. Statistical analysis was performed using Statistica version 10 including medical package. To verify the hypothesis of the normal distribution of numerical data the Shapiro-Wilk and Kolmogorov-Smirnov tests were used. To analyze the distribution of particular genotypes in both groups, Pearson's chi^2^ test was used. To establish the effect of CB1 receptor genotypes on clinical phenotypes in both groups we used multivariate stepwise regression analysis.

The differences were considered statistically significant at a *P* value ≤0.05.

## 3. Results

Characteristics of study and control groups are shown in Tables 1, 2, and 3.

### 3.1. Frequency of NAFLD in Study Population

NAFLD was significantly more frequent in PCOS women than in the control group; it was present in 92 women (69.7%) with PCOS versus 40 women from the control group (30.3%) (*P* < 0.00028, OR = 2.414, and RR = 1.662) (Table 5).

### 3.2. *CNR1* Polymorphisms

Genetic analysis was performed in all studied groups: 173 women with PCOS and 125 women from the control group. Frequency of the 6 assessed polymorphisms of the* CNR1* gene were compared in PCOS + NAFLD versus PCOS − NAFLD and in control + NAFLD versus control − NAFLD. No statistically significant differences in* CNR1* genetic variants were observed in the control group. In the PCOS group we observed significant differences in frequency of assessed polymorphisms: the G allele of rs806381 (*P* < 0.025) and the GG genotype of rs10485170 (*P* < 0.03) were more frequent in PCOS women with NAFLD than in PCOS women without NAFLD. There was a higher frequency of TT genotype of rs6454674 in PCOS women with NAFLD but this association was not significant (*P* = 0.059). Frequencies of polymorphisms of* CNR1* are shown in Table 6.

In stepwise regression analysis the GG genotype of rs806381 is associated with PCOS + NAFLD phenotype and increases its risk (OR = 2.914; *P* = 0.016). The GA genotype of rs806381 reduces the risk of PCOS + NAFLD phenotype by approximately 70% (*P* = 0.002). GT genotype of rs6454674 significantly increases the risk of PCOS + NAFLD phenotype (OR = 2.6; *P* = 0.0130). There was no association between GG genotype of rs6454674 and phenotypes in the PCOS group because of the small number of carriers of GG genotype in both subgroups (only 3 cases in PCOS − NAFLD versus 10 cases in PCOS + NAFLD) (results shown in Table 7).

## 4. Discussion

In our study we observed significantly more frequent NAFLD in the group of PCOS women (*P* < 0.00028, OR = 2.414; RR = 1.662). According to many data, there is a higher proportion of NAFLD among women with PCOS and PCOS features in women with NAFLD: 71% of NAFLD women matched the Rotterdam criteria for PCOS and 41% of PCOS women had concomitant NAFLD, whereas the incidence of NAFLD in the weight- and age-matched non-PCOS control group was only 19% [7]. According to our study, the risk of NAFLD in the PCOS group was 2.5 times higher than that of the control group. The first evidence for the association of NAFLD and PCOS was reported in 2005 [44]. The prevalence of NAFLD in PCOS women may occur irrespectively of obesity, as reported by Gambarin-Gelwan et al. [45]. Not only components of MS but also decreased sex hormone binding globulin (SHBG) and increased free androgen index (FAI) were linked to NAFLD [9, 46]. Therefore hyperandrogenism can be implicated with increased prevalence of NAFLD in PCOS women. Data on the association of these two disorders has yielded conflicting results. Markou et al. did not reveal any association between these two conditions in young lean women [47]. Despite this controversy, early detection of NAFLD in PCOS women is very important because early intervention may decrease or eliminate the possibility of liver disease progression and, similarly, women with NAFLD should be routinely screened for presence of features of PCOS.

Taking into account possible involvement of the EC in NAFLD etiopathogenesis and the impact of the EC on ovaries, we investigated the link between* CNR1* genotypes and NAFLD as well as PCOS. So far, one study concerning* CNR1* polymorphism and NAFLD has been conducted. This study revealed that a wild variant of rs1049353 was associated with worse metabolic profile, and carriers of the A allele had a lower grade of liver fibrosis evaluated by liver biopsy. Other studies were related to the connection of common genetic variants of* CNR1* with metabolic risk factors but data are contradictory. Numerous studies have revealed that polymorphisms of* CNR1*, rs11049353, rs12720071, rs806381, rs10485170, rs6454674, and rs2023239, were associated with features of MS such as increased BMI and waist circumference [11, 14, 48]. The G allele of rs1049353 polymorphism was associated with a decreased level of adiponectin and GG homozygotes were overweight or obese [11, 48, 49]. There was also an association of rs1049353 with obesity, IR, and adipocytokines in a group of women with obesity and with fat distribution and abdominal adiposity in men [50–52]. An association of rs1049353 with waist circumference, waist-hip ratio (WHR), and BMI was also revealed [14]. Other studies observed no association between this polymorphism and obesity, CV risk factors, or adipocytokines [51, 53, 54]. According to the study of Peeters et al. there was no significant association of this polymorphism with obesity although the G allele was related to increased waist circumference and WHR [51]. In our study we found no association of rs1049353 with NAFLD in either controls or PCOS women. In the study of Benzinou et al. rs806381 was associated with obesity and BMI [55]. According to one study, it was also associated with TG level but not with BMI or WHR [56]. In our study the G allele of rs806381 (*P* < 0.025) and the GG genotype of rs10485170 (*P* < 0.03) were significantly more frequent in women with PCOS and NAFLD in comparison to PCOS women without NAFLD. We also observed higher frequency of the TT genotype of rs6454674 in PCOS women with NAFLD but this association was not significant (*P* = 0.059). This might indicate the possible role of these polymorphisms in the development of NAFLD, but this association was significant only in the PCOS group. Explanation of such relationships only in PCOS women is difficult. It might result from other epigenetic (e.g., environmental) influences and different hormonal milieu which can interact with genetic factors. According to these results, we used stepwise regression to establish the effect of CB1 genotypes (rs6454674, rs806381, and rs10485170) on clinical phenotypes in both groups: PCOS + NAFLD versus PCOS − NAFLD (Table 7). We demonstrated that the GG genotype of rs806381 is associated with PCOS + NAFLD phenotype and increases its risk (OR = 2.914; *P* = 0.016). The GA genotype of rs806381 reduces the risk of PCOS + NAFLD phenotype by approximately 70% (*P* = 0.002). In the case of rs6454674, the GT genotype significantly increases the risk of PCOS + NAFLD phenotype (OR = 2.6; *P* = 0.0130). These results might suggest that the G allele of rs806381 could be a risk allele for NAFLD in PCOS. Unfortunately, we did not find any association between the GG genotype of rs6454674 and phenotypes in the PCOS group, because of the small number of carriers of the GG genotype in both subgroups (only 3 cases in PCOS − NAFLD versus 10 cases in PCOS + NAFLD). These results might indicate a possible connection between* CNR1* variants and etiopathology of NAFLD in PCOS women, which is complex and multifactorial. It is possible that NAFLD not only is a simple complication of MS, IR, and hyperandrogenism but also is related to genetic polymorphisms of the EC which may affect its function. We observed no significant associations between rs12720071 or rs806368 and NAFLD frequency in both study groups. Rs12720071 can influence body fat mass and fat distribution in men [57]. Carriers of the G allele had a higher level of total body fat and central fat deposition [57]. Rs806368 was associated with obesity but it did not remain significant after accounting for multiple testing [58]. Results of studies are conflicting. No associations between rs806381, rs10485170, rs6454674, and rs2023239 polymorphisms and anthropometric variables were observed in a population of postmenopausal women from Poland [59, 60]. To our knowledge, this is the first study evaluating the link between genetic variants of* CNR1* and NAFLD in PCOS women.

A limitation of our study was the method used to evaluate liver steatosis. Although ultrasonography is the most widely used method for detecting liver steatosis, with an acceptable level of sensitivity, it is less sensitive than liver biopsy, which can detect 5% of fat infiltration within the liver. Another limitation was the number of genetic variants of* CNR1* assessed in our study. We assessed only six common polymorphisms of* CNR1* but there are also several other polymorphisms that can be associated with adverse metabolic and cardiovascular profiles, such as rs2023239, rs806378, rs806365, and rs10485179, whose relationship with obesity, type 2 DM, and IR was revealed.

In summary, our study showed significantly more frequent NAFLD in the PCOS group, which indicates that it is reasonable to carry out an ultrasound evaluation of the liver in all women with PCOS. We also observed significantly higher frequency of polymorphic variants of* CNR1* (G allele of rs806381 and GG genotype of rs10485170) in women with PCOS and NAFLD in comparison with PCOS women without NAFLD. This association was found only in the PCOS group, which might result from the interaction between genetic factor and hormonal milieu. The stepwise regression analysis revealed that the G allele of rs806381 could be a risk allele for NAFLD in PCOS women. These results might indicate the potential impact of genetic variants of* CNR1* on NAFLD etiopathology in PCOS. We did not find any relationship between the GG genotype of rs6454674 and NAFLD in PCOS possibly because of the small number of carriers of this genotype. Further studies on EC's impact on metabolic complications in PCOS with a larger number of cases are needed.

## Figures and Tables

**Table 1:** Characteristics of study and control groups—anthropometric parameters.

	Age	Weight (kg)	BMI (kg/m^2^)	Waist (cm)	Hip (cm)	WHR
Control − NAFLD	27.6 ± 6.6	63.3 ± 11.2	22.7 ± 3.6	78.4 ± 10.6	100.3 ± 14.7	0.8 ± 0.1
Control + NAFLD	27.7 ± 5.8	72.2 ± 18.8	26.4 ± 6.3	85.3 ± 16.6	105.3 ± 12.6	0.8 ± 0.1
PCOS − NAFLD	24.1 ± 4.4	65.4 ± 14.8	24.5 ± 8.0	79.6 ± 13.0	99.2 ± 9.4	0.8 ± 0.1
PCOS + NAFLD	25.3 ± 5.82	80.6 ± 22.4	28.7 ± 7.4	91.1 ± 18.6	106.9 ± 12.3	0.8 ± 0.1

Data are presented as mean ± SD.

**Table 2:** Characteristics of study group (PCOS group).

	PCOS + NAFLD	PCOS − NAFLD	Whole group (PCOS)
GOT (U/L)	24.06 ± 11.22	22.13 ± 8.36	23.24 ± 10.13
GPT (U/L)	27.17 ± 18.09	20.26 ± 14.35	24.25 ± 16.92
GGTP (U/L)	30.37 ± 21.83	21.74 ± 13.89	26.66 ± 19.27
Bilirubin (mg/dL)	0.677 ± 0.364	0.694 ± 0.430	0.684 ± 0.391
Triglycerides (mg/dL)	120.00 ± 70.18	94.60 ± 58.52	109.30 ± 66.53
Total cholesterol (mg/dL)	191.00 ± 36.22	185.30 ± 37.39	188.60 ± 36.72
LDL cholesterol (mg/dL)	113.30 ± 32.27	104.10 ± 28.93	109.50 ± 31.17
HDL cholesterol (mg/dL)	53.63 ± 16.3	63.68 ± 19.63	57.88 ± 18.42
Mean glucose level (mg/L)	113.40 ± 23.54	102.40 ± 24.97	108.70 ± 24.7
Mean insulin level (*μ*IU/mL)	60.46 ± 43.89	37.40 ± 20.56	50.59 ± 37.52
Testosterone (ng/mL)	0.653 ± 0.295	0.57 ± 0.322	0.619 ± 0.308
SHBG (mmol/L)	34.17 ± 19.63	46.06 ± 29.42	39.18 ± 24.87
FAI value	9.394 ± 8.484	6.10 ± 5.151	8.008 ± 7.434

Data are presented as mean ± SD.

**Table 3:** Characteristics of control group.

	Control + NAFLD	Control − NAFLD	Whole group (control)
GOT (U/L)	20.08 ± 7.63	18.95 ± 3.95	19.51 ± 6.06
GPT (U/L)	20.23 ± 11.54	17.19 ± 7.47	18.70 ± 9.79
GGTP (U/L)	21.03 ± 9.45	17.76 ± 5.59	19.38 ± 7.89
Bilirubin (mg/dL)	2.605 ± 0.51	1.325 ± 0.78	1.97 ± 0.66
Triglycerides (mg/dL)	83.39 ± 53.37	71.63 ± 27.21	77.51 ± 42.60
Total cholesterol (mg/dL)	181.70 ± 37.33	184.20 ± 39.03	183.00 ± 38.09
LDL cholesterol (mg/dL)	108.60 ± 37.18	106.60 ± 31.34	107.60 ± 34.26
HDL cholesterol (mg/dL)	66.02 ± 21.72	66.65 ± 14.83	66.33 ± 18.52
Mean glucose level (mg/L)	101.2 ± 19.53	95.8 ± 16.63	98.4 ± 18.25
Mean insulin level (*μ*IU/mL)	40.58 ± 35.71	32.82 ± 21.95	36.67 ± 29.72
Testosterone (ng/mL)	0.397 ± 0.154	0.368 ± 0.163	0.382 ± 0.159
SHBG (mmol/L)	49.87 ± 30.17	58.66 ± 25.99	54.48 ± 28.28
FAI value	3.99 ± 3.33	2.55 ± 1.63	3.23 ± 2.67

Data are presented as mean ± SD.

**Table 4:** Sequences of *CNR1* primers.

Polymorphism	Forward primer (3′-5′)	Reverse primer (3′-5′)
A3813G (rs12720071)	GATGAAGGCTCAGGGTGCTAGAGG	TAGTGCTGTCAGCCCCATTGTCCC
G1422A (rs1049353)	CCTGCGACACGCTTTCCGGA	CTGCCAGGGAGGCATCAGGC
A4895G (rs806368)	GAGACCACCCATATCATGCACACA	AACTCTGATCCCCAGTAGGCCTAG
rs806381	CATGAGCCATGAGGTTTTCT	CATTTGAAGGCCTGTAACTT
rs10485170	TTAACCAATG GTTCATCGTC	ATGTGGTTCTCAGGCATCAG
rs6454674	ATGGAGCCTGTCCTTTAGGT	TATCCAGGAATGCTGCAAAA

**Table 5:** Frequency of NAFLD in study and control groups.

	NAFLD	Without NAFLD
PCOS	92 (69.70%)	81 (48.80%)
Control	40 (30.30%)	85 (51.20%)

*P* < 0.00028, OR = 2.414, and RR = 1.662.

**Table 6:** Frequency of *CNR1* polymorphisms in study and control groups.

	A3813G (rs12720071)			*P* (chi^2^)	A4895G (rs806368)			*P* (chi^2^)
	A/A	A/G	G/G		T/T	C/T	C/C	
PCOS + NAFLD	64.41% (38)	68.42% (13)	50% (13)	0.35 (2.04)	58.82% (40)	57.69% (15)	87.5% (7)	0.27 (2.6)
PCOS − NAFLD	35.59% (21)	31.58% (6)	50% (13)		41.18% (28)	42.31% (11)	12.5% (1)	
Control + NAFLD	48.48% (32)	28.57% (4)	66.67% (4)	0.24 (2.89)	46.3% (25)	46.88% (15)	100% (1)	0.57 (1.14)
Control − NAFLD	51.52% (34)	71.43% (10)	33.33% (2)		53.7% (29)	53.13% (17)	0.00% (0)	

	G1422A (rs1049353)			*P* (chi^2^)	rs806381			*P* (chi^2^)
	A/A	G/A	G/G		A/A	G/A	G/G	

PCOS + NAFLD	48% (12)	69.7% (23)	64.58% (31)	0.21 (3.05)	72% (18)	37.5% (9)	66.67% (28)	0.025 (7.36)
PCOS − NAFLD	52% (13)	30.3% (10)	35.42% (17)		28% (7)	62.5% (15)	33.33% (14)	
Control + NAFLD	33.33% (4)	50% (14)	48.98% (24)	0.58 (1.07)	45% (18)	50% (21)	44.44% (8)	0.88 (0.26)
Control − NAFLD	66.67% (8)	50% (14)	51.02% (25)		55% (22)	50% (21)	55.56% (10)	

	rs10485170			*P* (chi^2^)	rs6454674			*P* (chi^2^)
	A/A	A/G	G/G		T/T	G/T	G/G	

PCOS + NAFLD	61.9% (52)	45.45% (5)	75% (6)	0.03 (7.04)	69.39% (34)	48.89% (22)	76.92% (10)	0.06 (5.62)
PCOS − NAFLD	38.1% (32)	54.55% (6)	25% (2)		30.61% (15)	51.11% (23)	23.08% (3)	
Control + NAFLD	50% (41)	42.86% (9)	0%	0.56 (0.34)	48.98% (24)	50% (20)	42.86% (6)	0.89 (0.22)
Control − NAFLD	50% (41)	57.14% (12)	0%		51.02% (25)	50% (20)	57.14% (8)	

PCOS + NAFLD: women with polycystic ovary syndrome and nonalcoholic fatty liver disease.

PCOS − NAFLD: women with polycystic ovary syndrome without nonalcoholic fatty liver disease.

Control + NAFLD: women from control group having nonalcoholic fatty liver disease.

Control − NAFLD: women from control group without nonalcoholic fatty liver disease.

**Table 7:** Stepwise regression analysis.

	*P*	OR	CI OR 95%	CI OR 95%
rs806381-GA	0.002	0.308	0.145	0.654
rs806381-GG	0.016	2.914	1.220	6.960
rs6454674-GT	0.013	2.628	1.225	5.636
